# Effect of Patellar Denervation on Anterior Knee Pain and Knee Function in Total Knee Arthroplasty without Patellar Resurfacing: A Meta‐Analysis of Randomized Controlled Trials

**DOI:** 10.1111/os.12815

**Published:** 2020-10-28

**Authors:** Yuhang Wang, Wei Feng, Junting Zang, Hang Gao

**Affiliations:** ^1^ Day Care Unit The First Hospital of Jilin University Changchun China; ^2^ Department of Orthopaedics The First Hospital of Jilin University Changchun China

**Keywords:** Anterior knee pain, Clinical outcome, Patellar denervation, Total knee arthroplasty

## Abstract

**Objective:**

The aim of the present study was to evaluate the effect of patellar denervation (PD) in preventing anterior knee pain (AKP) and improving knee function after total knee arthroplasty (TKA) without patellar resurfacing, and to help surgeons decide whether or not to use PD in TKA.

**Methods:**

The electronic databases of Pubmed, Embase, Cochrane, Web of Science, and Scopus were searched for all randomized controlled trials (RCT) comparing the outcomes of PD and no patellar denervation (NPD) in TKA without patellar resurfacing. Primary outcomes were incidence of AKP, visual analogue scale for pain (VAS), and patellar score (PS). Secondary outcomes were Knee Society Score (KSS), the Western Ontario and McMaster Universities Osteoarthritis Index (WOMAC), the Oxford Knee Score (OKS), knee range of motion (ROM), and complications.

**Results:**

A total of nine RCT met the inclusion criteria. On meta‐analysis, PD significantly reduced the incidence of AKP (odds ratio 0.49; 95% confidence interval [CI] 0.26 to 0.92), reduced the VAS (weighted mean difference [WMD] −0.57; 95% CI −1.02 to −0.11), and improved the WOMAC (WMD −4.63; 95% CI −6.49 to −2.77) and the ROM (WMD 9.60; 95% CI 0.39 to 18.81) during the follow‐up within 12 months. In addition, PD improved the PS (WMD 1.01; 95% CI 0.65 to 1.38), KSS (WMD 1.12; 95% CI 0.10 to 2.14), and the WOMAC (WMD −1.41; 95% CI −2.74 to −0.08) during the follow‐up after 12 months.

**Conclusion:**

Patellar denervation could significantly reduce the VAS and the incidence of AKP in the early stages after TKA as well as improve the clinical outcomes in terms of the PS, the WOMAC, the KSS, and the ROM. This study demonstrates that PD is a safe and recommendable technique that could be routinely performed in TKA.

## Graphical abstract

To evaluate the effect of PD in preventing AKP and improving knee function after TKA without patellar resurfacing and to help surgeons decide whether or not to use PD in TKA. The electronic databases of Pubmed, Embase, Cochrane, Web of Science and Scopus were searched for all randomized controlled trials (RCT) comparing the outcomes of PD and no patellar denervation (NPD) in TKA without patellar resurfacing. PD could significantly reduce the VAS and the incidence of AKP in the early stages after TKA, and improve the clinical outcomes in terms of the PS, the WOMAC, the KSS, and the ROM. This study demonstrates that PD is a safe and recommendable technique that could be routinely performed in TKA.

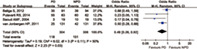

## Introduction

Total knee arthroplasty (TKA) is an effective surgical procedure to relieve pain and improve knee function in patients with osteoarthritis. However, anterior knee pain (AKP) after TKA could negatively influence patient satisfaction and knee function^1^. AKP has been reported in 4% to 49% of patients undergoing primary TKA without patellar resurfacing^2^, ^3^. In theory, patellar denervation (PD) with electrocautery could reduce the incidence of AKP by blocking superficial sensory nerves around the patella and improve knee mobility^4^.

Nevertheless, no consistent conclusion regarding PD has been reached in research published to date. Motififard *et al*.^5^ and Alomran^6^, respectively, reported that PD with electrocautery could prevent AKP in the early postoperative period and improve postoperative knee function. In contrast, Kwon *et al*.^1^ found that PD provided no benefit in a randomized controlled trial. Furthermore, recent meta‐analyses also failed to reach any consensus^7^, ^8^. No meta‐analysis, to our knowledge, has been undertaken investigating the effect of PD with different follow‐up (FU) durations after TKA. Thus, we previously grouped the outcomes and extracted the data according to FU to evaluate the outcomes in different periods after operation.

This meta‐analysis study aims to assess the effect of PD in preventing AKP and improving knee function after TKA, and to help surgeons decide whether or not to use PD in TKA procedures.

## Methods

Our study was performed according to the PRISMA statement criteria and was registered with the International Prospective Register of Systematic Reviews (PROSPERO) (Registration number CRD42019147808).

### *Search Strategy*

We searched the literature for all randomized controlled trials (RCT) comparing PD and no patellar denervation (NPD) using the electronic databases of Pubmed, Embase, Cochrane, Web of Science, and Scopus in January 2020. No time limitation or language was applied. Moreover, we conducted a manual search for all potentially eligible studies in the reference lists of all review articles and reports. The following search strategy was used: (((((“Denervation”[Mesh]) OR ((Denervation*[Title/Abstract]) OR Neurectom*[Title/Abstract]))) OR ((((“Electrocoagulation”[Mesh]) OR Electrocautery[Title/Abstract]) OR Thermocoagulation[Title/Abstract]) OR Galvanocautery[Title/Abstract]))) AND (((“Arthroplasty, Replacement, Knee”[Mesh]) OR knee arthroplasty[Title/Abstract]) OR knee replacement[Title/Abstract]).

### *Inclusion and Exclusion Criteria*

Two authors respectively screened the titles and abstracts of all studies. A discussion would be organized to resolve any disagreement, and a third author would make a final judgment if no consensus was reached. Inclusion criteria: (i) Participants: adult patients undergone TKA and minimum 3‐month FU; (ii) Interventions: PD was used in the surgery; (iii) Comparisions: NPD was used in the surgery; (iv) Outcome measure: incidence of AKP, clinical outcome scores, and complications; and (v) Study design: RCT. Exclusion criteria: Studies that included patients with knee deformity greater than 15°, a history of patellar operation, and patellar fracture or dislocation without operation.

### *Data Collection And Extraction*

To assess the effect of PD with different FU after TKA, we specified two subgroups according to FU (short‐term [ST]: FU ≤12 months, and long‐term [LT]: FU > 12 months). According to the two subgroups, two independent authors extracted data from eligible studies using a standardized data collection form. Any discrepancy was resolved by discussion, or a senior author would make a final judgment if no consensus was reached. The following items were collected from each study: the first author's name, publication date, country of origin, study design, number of patients and knees, gender percentage, age, body mass index (BMI), diagnosis, depth of electrocautery, surgical approach, use of cement, follow‐up duration, and clinical outcome in PD and NPD groups. Primary outcomes were incidence of AKP, visual analogue scale for pain (VAS), and patellar score (PS). Secondary outcomes were the Knee Society score (KSS), the Western Ontario and McMaster Universities Osteoarthritis Index (WOMAC), the Oxford knee score (OKS), knee range of motion (ROM), and complications.

#### *Incidence of Anterior Knee Pain*

Anterior knee pain after TKA could negatively influence patient satisfaction and knee function. To investigate the effect of PD in TKA, the incidence of AKP was collected according to the patient‐reported qualitative and quantitative data.

#### *Visual Analogue Scale for Pain*

The VAS is a commonly used scoring criteria of pain, with pain rated on a scale of 0 to 10. We collected the VAS data from the patient‐reported qualitative and quantitative data.

#### *Patellar Score*

The PS is a frequently‐used clinical score for evaluating the functions of patellofemoral and knee joints. According to the PS, the severity of AKP was graded as “none,” “mild,” “moderate,” and “severe.”

#### *Knee Society Score*

The KSS is a self‐administered questionnaire for assessing the function of the knee following knee surgeries or diseases. Subjective and objective domains were included in the KSS, which should be completed by both surgeons and patients. Higher scores indicate less pain and better function.

#### *Western Ontario and McMaster Universities Osteoarthritis Index*

The WOMAC is widely used to assess the function of knees or hips from the following three domains: pain, stiffness, and physical function. Higher scores indicate worse pain, stiffness, and physical function.

#### *Oxford Knee Score*

The OKS is used to assess the function of the knee from the following two domains: pain and physical function. There are five items in the pain domain and seven items in the physical function domain. Higher scores indicate worse pain and physical function.

#### *Knee Range of Motion*

The ROM refers to the angle of rotation of a joint during motion. The ROM was collected from medical records.

#### *Complications*

Infection, stiffness, deep vein thrombosis, patellar osteonecrosis, and loosening of implants were considered as complications, and the data were collected from medical records.

### *Risk‐of‐Bias and Quality Assessments*

Two reviewers respectively assessed the potential risk‐of‐bias of all eligible studies using the Cochrane Collaboration's Tool for Assessing Risk of Bias^9^ in seven domains: random sequence generation, allocation concealment, blinding of participants and personnel, blinding of outcome assessment, incomplete outcome data, selective reporting, and other sources of bias. The quality of evidence of each outcome was graded using the Grading of Recommendation, Assessment, Development and Evaluation (GRADE) approach.

### *Statistical Analysis*

The statistical analysis was performed using RevMan software (version 5.2; the Nordic Cochrane Centre, Copenhagen, Denmark) and STATA (version 13.1; StataCorp, TX, USA). The odds ratio (OR) with 95% confidence intervals (CI) was calculated for dichotomous data. The weighted mean difference (WMD) with a 95% CI was calculated for continuous data. The *I* ^2^ statistic and the *χ^2^*‐test were calculated to test the statistical heterogeneity. An *I* ^2^ value ≤50% and a *P*‐value >0.1 indicated no statistical heterogeneity between studies. A fixed‐effects model would be used for meta‐analysis when there was no statistical evidence of heterogeneity, or else a random‐effects model would be adopted. The sensitivity analysis was performed to explore the potential sources of heterogeneity.

## Results

### *Literature Search Results and Study Characteristics*

**Fig. 1:**
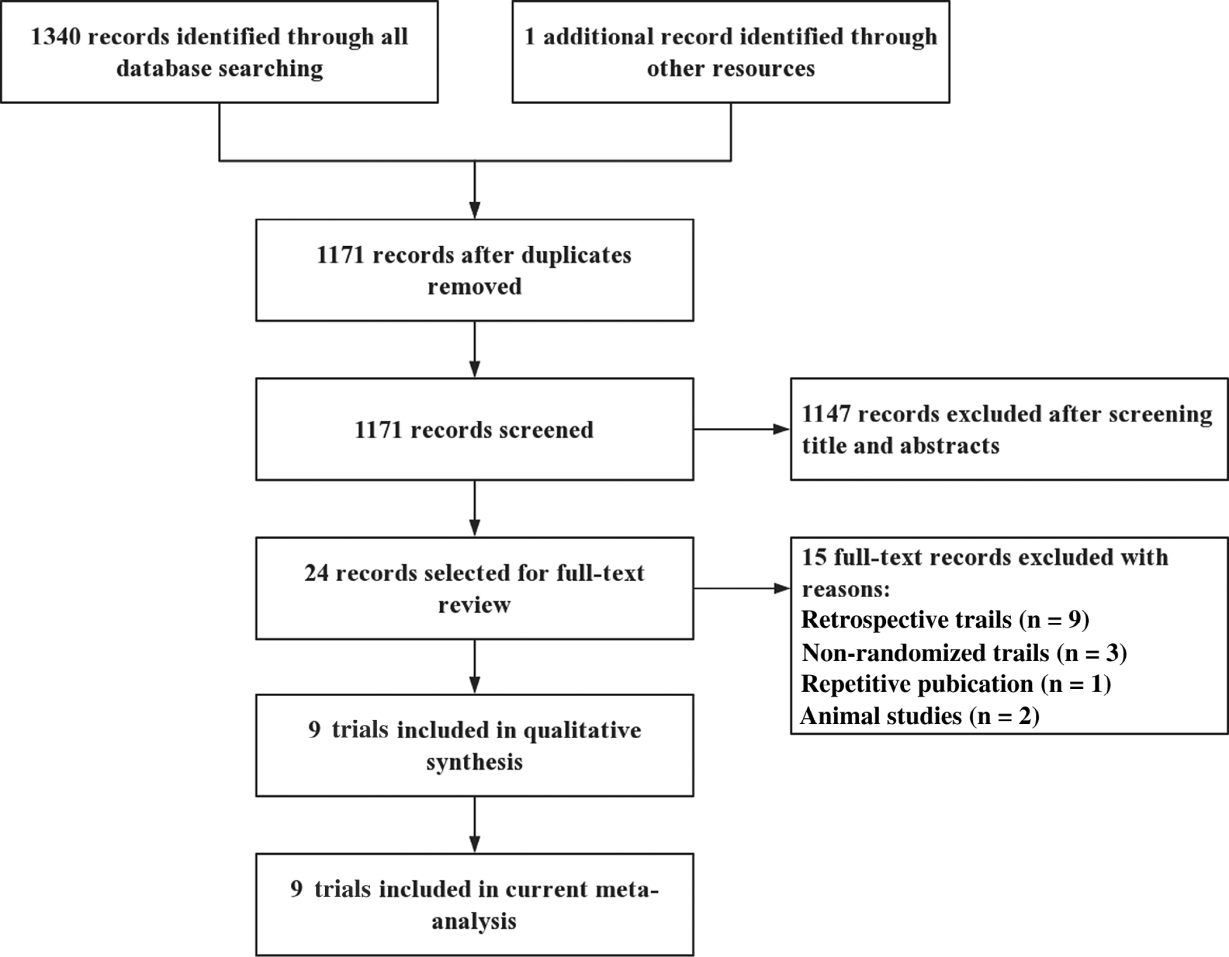
Flow diagram shows the process of study indentification, inclusion, and exclusion.

We identified 1341 records using the literature search strategy. After duplicates had been removed, the titles and abstracts of 1171 records were screened, and 24 trials were selected for full‐text review. Finally, nine studies met the inclusion criteria and were included in the current meta‐analysis^1^, ^4^, ^6^, ^10^, ^11^, ^12^, ^13^, ^14^, ^15^. A total of 1195 patients (1267 knees) with an age range from 42 to 91 years were included, of which 598 patients (634 knees) were treated with PD and 597 patients (633 knees) were treated with NPD. The PD group contained 29.6% male and 70.4% female patients, and the NPD group contained 32.4% male and 67.6% female patients. In the PD group, patellar denervation was performed following knee arthroplasty. In the NPD group, no patellar denervation was performed. The mean length of FU was 28.6 months (range, 12 to 60 months). The process of literature searching and study selection is shown in Fig. 1. The characteristics of the included studies are presented in Table 1.

**TABLE 1:** Characteristics of the included studies

Reference number	Author	Year	Country	Knee (*n*)		Gender (F/M)		Age		Diagnosis	Depth of electrocautery	Approach	Cement	Patellar management in control group	FU
				PD	NPD	PD	NPD	PD	NPD						
1	Alomran A	2015	Saudi Arabia	92	92	NA	NA	NA	NA	OA	1 mm	MP	Yes	Osteophytes and anterior fat pad were removed	3 years
2	Altay MA	2012	Turkey	35	35	26/9	26/9	68	68	NA	2–3 mm	MV	Yes	Removal of all osteophytes	36 months
3	Baliga S	2012	UK	91	94	40/51	42/52	69	69.2	OA	NA	MP	Yes	Osteophytes producing impingement or poor patellar tracking were removed	12 months
4	Kwon SK	2015	Korea	50	50	50/0	50/0	66.3	67.0	NA	1 mm	MP	NA	Debridement of osteophytes from the patella	5 years
5	Pulavarti RS	2014	UK	63	63	32/31	36/27	69.9	69.8	OA	NA	MP	Yes or Not	excision of osteophytes around the patella	24 months
6	Saoud AMF	2004	Egypt	19	19	NA	NA	NA	NA	NA	1 mm	NA	NA	NA	9–12 months
7	Van Jonbergen HP	2011	Netherlands	131	131	95/36	84/47	71	72	OA	≤1 mm	MP	Yes	Osteophytes were removed	1 year
8	Van Jonbergen HP	2014	Netherlands	103	99	75/28	65/34	70	71	OA	≤1 mm	MP	Yes	Osteophytes were removed	3.7 years
9	Yim SJ	2012	Korea	50	50	50/0	50/0	70.2	70.2	OA	NA	MP	Yes	NA	21 months

FU, follow‐up duration; MP, medial parapatellar approach; MV, midvastus approach; NA, not available; NPD, no patellar denervation; OA, osteoarthritis; PD, patellar denervation.

### *Risk‐of‐Bias and Study Quality*

**Fig. 2:**
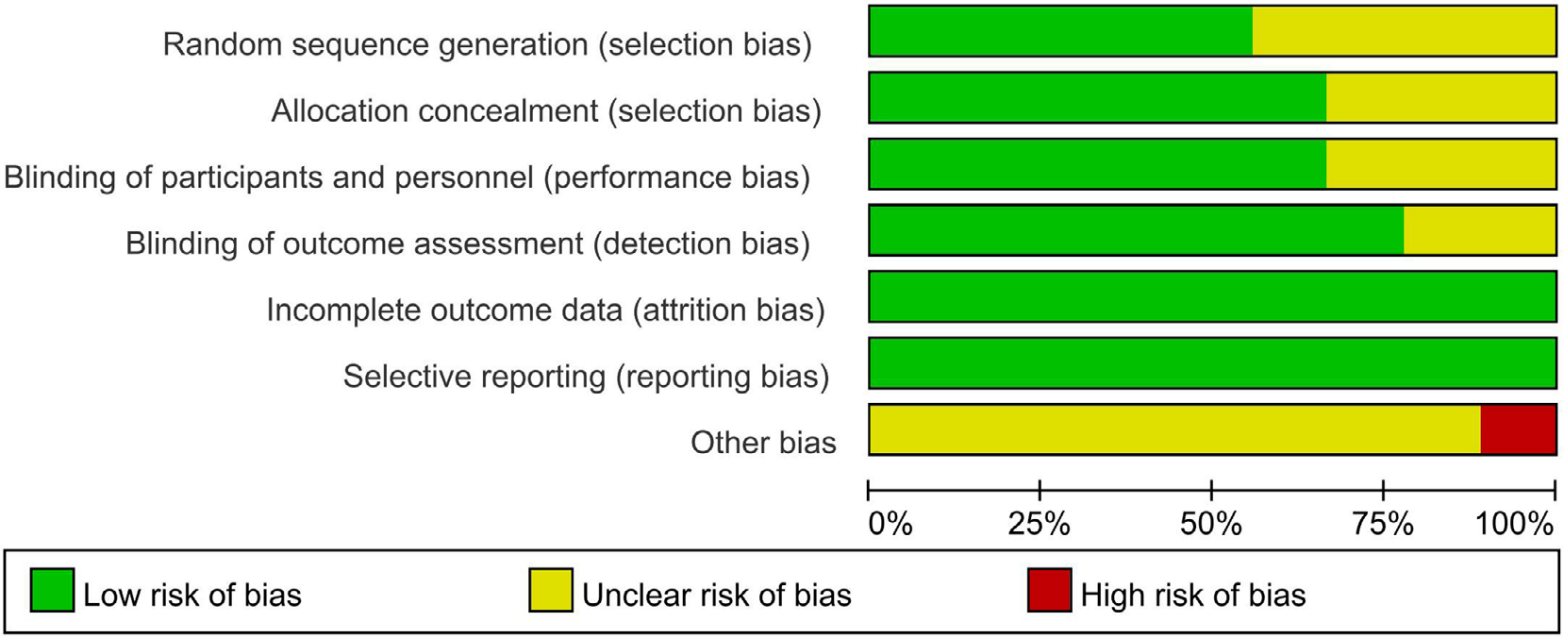
Risk of bias graph.

**Fig. 3:**
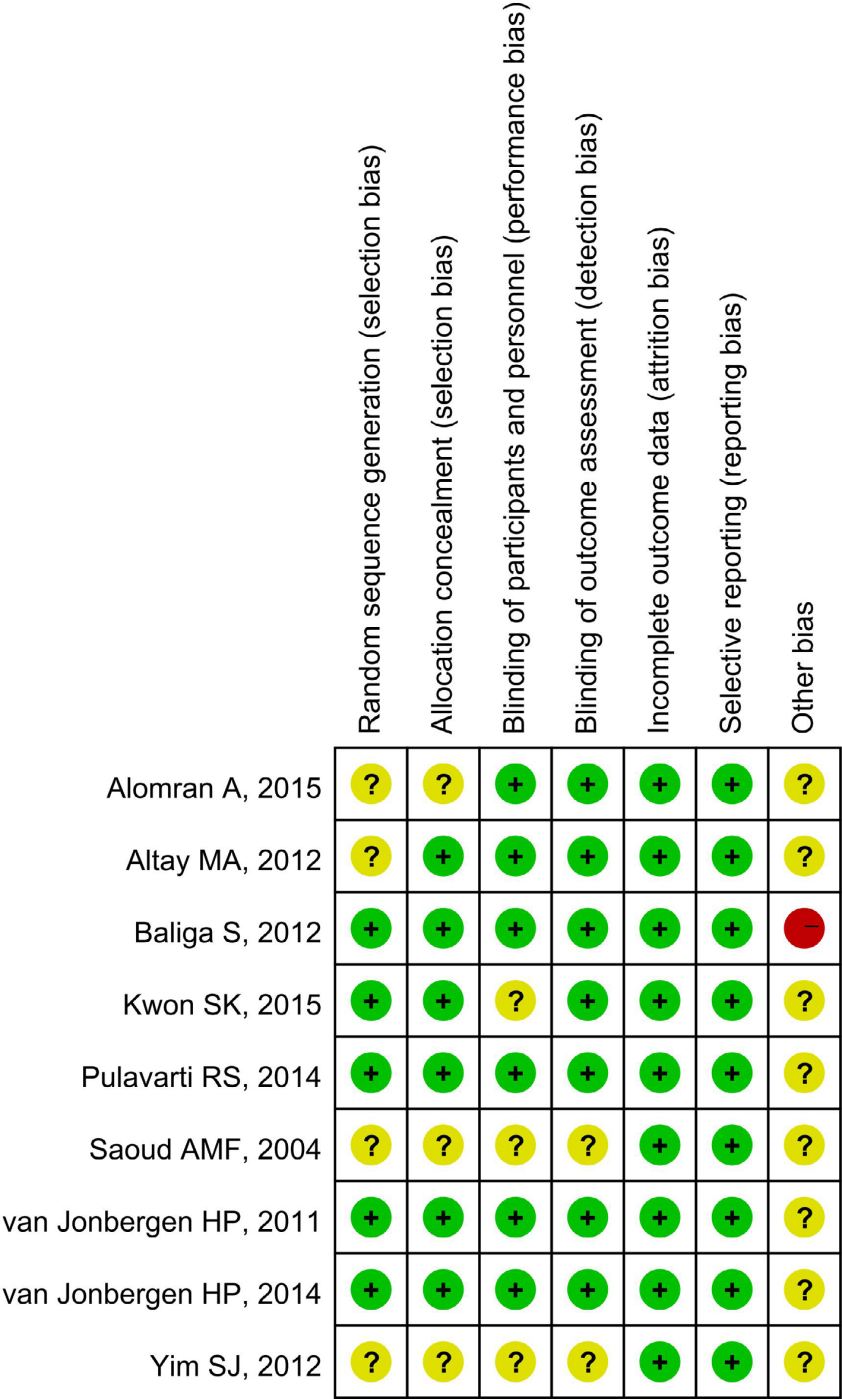
Risk of bias for the included studies. (“+” indicates a low risk of bias, “‐” indicates a high risk of bias, and “?” indicates unclear or unknown risk of bias).

The result of risk‐of‐bias is presented in Figs 2 and 3. Five^1^, ^4^, ^10^, ^13^, ^14^ of the nine included studies conducted and described randomization fairly well. Six studies^1^, ^4^, ^10^, ^12^, ^13^, ^14^ described the method of allocation concealment. Blinding of participants was clearly described in six studies^4^, ^6^, ^10^, ^12^, ^13^, ^14^. Blinding of outcome assessment was illustrated in seven studies^1^, ^4^, ^6^, ^10^, ^12^, ^13^, ^14^. All nine included studies^1^, ^4^, ^6^, ^10^, ^11^, ^12^, ^13^, ^14^, ^15^ presented complete outcome data, and all the outcomes planned previously were reported, suggesting low risk of bias with respect to attrition bias and reporting bias. One study^13^ reported funding support, while no other bias was detected. The quality of the included studies was evaluated using the GRADE approach (Table 2).

**TABLE 2:** Quality assessment of studies

Subgroup	Outcomes	Quality assessment								Quality
		Risk of bias	Inconsistency	Indirectness	Imprecision	Publication bias	Large effect	Plausible confounding would change the effect	Dose–response gradi	
ST	Incidence of AKP	Serious	No	No	No	Undetected	No	No	No	Moderate
	VAS	Serious	No	No	No	Undetected	No	No	No	Moderate
	PS	No	No	No	No	Undetected	No	No	No	High
	KSS	No	No	No	No	Undetected	No	No	No	High
	KSSFS	No	No	No	No	Undetected	No	No	No	High
	WOMAC	No	No	No	No	Undetected	No	No	No	High
	WOMAC ‐ function	No	No	No	No	Undetected	No	No	No	High
	OKS	Serious	Serious	No	No	Undetected	No	No	No	Low
	ROM	No	Serious	No	No	Undetected	No	No	No	Moderate
	Incidence of complications	Serious	No	No	No	Undetected	No	No	No	Moderate
LT	Incidence of AKP	No	No	No	No	Undetected	No	No	No	High
	VAS	No	Serious	No	No	Undetected	No	No	No	Moderate
	PS	No	No	No	No	Undetected	No	No	No	High
	KSS	No	No	No	No	Undetected	No	No	No	High
	KSSFS	No	No	No	No	Undetected	No	No	No	High
	WOMAC	No	No	No	No	Undetected	No	No	No	High
	WOMAC ‐ function	No	No	No	No	Undetected	No	No	No	High
	ROM	No	No	No	No	Undetected	No	No	No	High
	Incidence of complications	No	No	No	No	Undetected	No	No	No	High

AKP, anterior knee pain; FU, duration of follow‐up; KSS, knee society score; KSSFS, KSS function score; LT, long‐term (FU > 12 months); OKS, Oxford knee score; PS, Feller's pateller score; ROM, range of motion; ST, short‐term (FU ≤12 months); VAS, visual analogue scale; WOMAC, the Western Ontario and McMaster University Osteoarthritis index.

### *Primary Outcome*

The results of the meta‐analysis are presented in Table 3.

**TABLE 3:** Meta‐analysis results of outcomes in included studies

Subgroup	Outcomes	Number of studies	Number of knees			Overall effect			Heterogeneity *P*‐value (*I* ^2^)	Statistical method
			Total	PD	NPD	*P*‐value	OR/WMD	95% CI		
ST	Incidence of AKP	4	610	304	306	0.03*	0.49	0.26, 0.92	0.11 (50%)	OR (M‐H, random)
	Incidence of AKP^#^	3	425	213	212	0.0004*	0.40	0.24, 0.66	0.32 (12%)	OR (M‐H, fixed)
	VAS	2	310	154	156	0.01*	−0.57	−1.02, −0.11	0.76 (0%)	WMD (IV, fixed)
	PS	2	225	113	112	0.81	0.12	−0.83, 1.06	0.28 (15%)	WMD (IV, fixed)
	KSS	3	487	244	243	0.84	0.31	−2.82, 3.45	0.06 (65%)	WMD (IV, random)
	KSS^#^	2	387	194	193	0.09	1.92	−0.32, 4.17	0.93 (0%)	WMD (IV, fixed)
	KSSFS	2	387	194	193	0.14	2.33	−0.80, 5.47	0.85 (0%)	WMD (IV, fixed)
	WOMAC	2	446	223	223	< 0.00001*	−4.63	−6.49, −2.77	0.76 (0%)	WMD (IV, fixed)
	WOMAC ‐ function	2	362	181	181	0.09	−2.14	−4.59, 0.31	0.16 (49%)	WMD (IV, fixed)
	OKS	2	310	154	156	0.90	−0.18	−2.92, 2.57	0.12 (59%)	WMD (IV, random)
	ROM	2	309	155	154	0.04*	9.60	0.39, 18.81	< 0.00001 (96%)	WMD (IV, random)
	Incidence of complication	3	425	211	214	0.27	2.56	0.49, 13.44	NA	OR (M‐H, fixed)
LT	Incidence of AKP	4	605	306	299	0.06	0.51	0.25, 1.04	0.05 (63%)	OR (M‐H, random)
	Incidence of AKP^#^	3	421	214	207	0.10	0.67	0.42, 1.08	0.68 (0%)	OR (M‐H, fixed)
	VAS	2	189	96	93	0.22	−0.31	−0.82, 0.19	0.11 (62%)	WMD (IV, random)
	PS	4	399	196	203	< 0.00001*	1.01	0.65, 1.38	0.23 (31%)	WMD (IV, fixed)
	KSS	4	472	238	234	0.03*	1.12	0.10, 2.14	0.23 (30%)	WMD (IV, fixed)
	KSSFS	3	372	188	184	0.15	2.34	−0.84, 5.52	0.08 (60%)	WMD (IV, random)
	KSSFS^#^	2	302	153	149	0.33	0.75	−0.75, 2.26	0.77 (0%)	WMD (IV, fixed)
	WOMAC	2	302	153	149	0.04*	−1.41	−2.74, −0.08	0.87 (0%)	WMD (IV, fixed)
	WOMAC ‐ function	2	302	153	149	0.53	0.98	−2.10, 4.07	0.20 (39%)	WMD (IV, fixed)
	ROM	3	289	146	143	0.10	2.62	−0.52, 5.75	0.02 (76%)	WMD (IV, random)
	Incidence of complication	6	775	391	384	0.18	0.61	0.30, 1.24	0.55 (0%)	OR (M‐H, fixed)

#Sensitivity analysis. *Significant difference. AKP, anterior knee pain; CI, confidence interval; FU, duration of follow‐up; KSS, knee society score; KSSFS, KSS function score; LT, long‐term (FU >12 months); NA, not available; No, number; NPD, no patellar denervation; OKS, Oxford knee score; OR, odds ratio; PD, patellar denervation; PS, Feller's pateller score; ROM, range of motion; ST, short‐term (FU ≤12 months); VAS, visual analogue scale; WMD, weighted mean difference; WOMAC, the Western Ontario and McMaster University Osteoarthritis index.

#### *Incidence of Anterior Knee Pain*

**Fig. 4:**
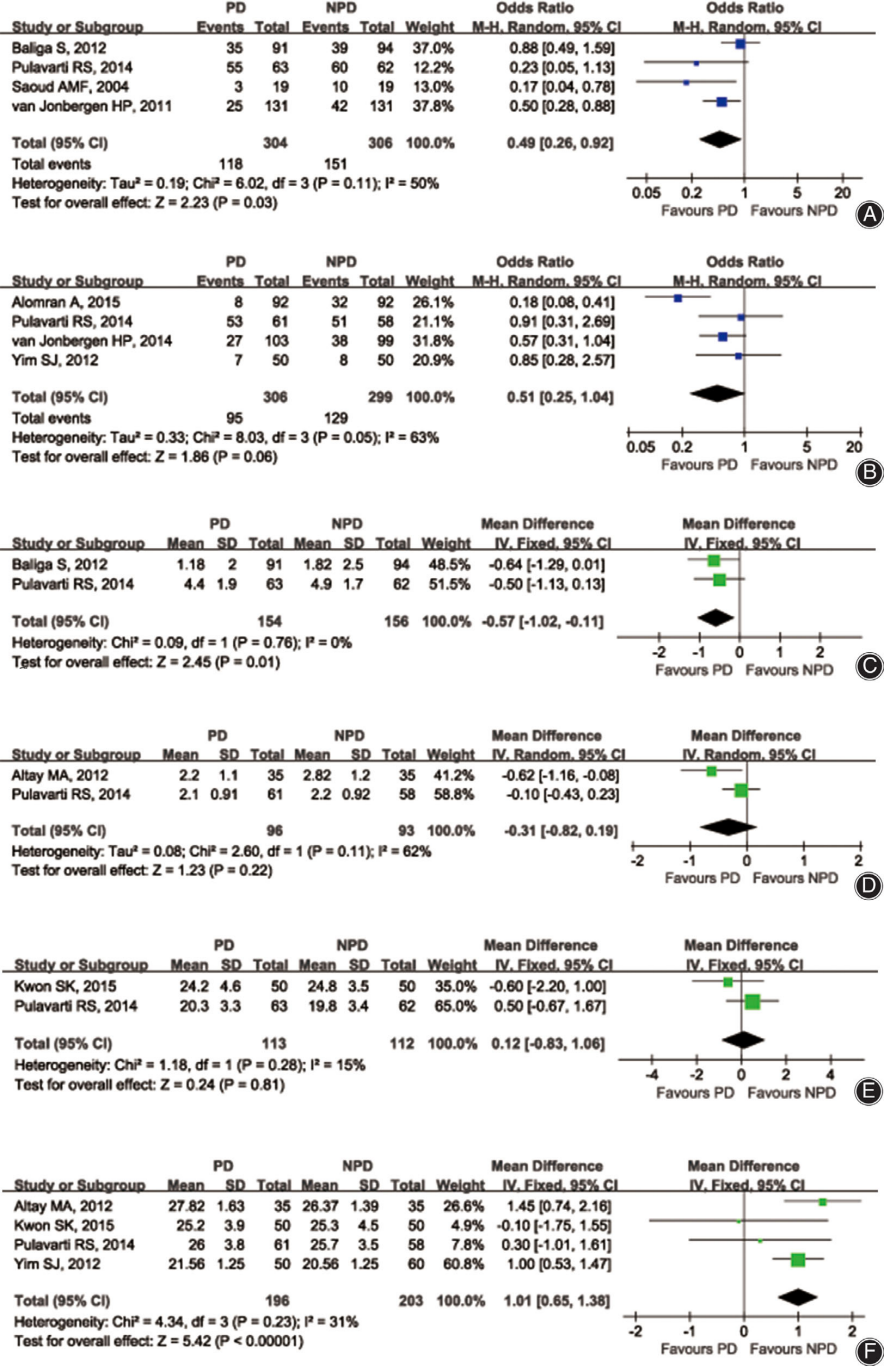
The forest plots show the overall outcome of the incidence of AKP (A and B), VAS (C and D), and PS (E and F). (AKP, anterior knee pain; CI, confidence interval; df, degrees of freedom; NPD, no patellar denervation; PD, patellar denervation; PS, Feller's pateller score; VAS, visual analogue scale).

In the ST subgroup, four studies^4^, ^13^, ^14^, ^15^ reported the incidence of AKP. The results indicated that PD significantly decreased the incidence of AKP (OR 0.49; 95% CI 0.26 to 0.92; *P* = 0.03; *n* = 610; *I* ^2^ = 50%; Fig. 4A). Similar statistical results were obtained with a sensitivity analysis, suggesting the stability of the result of the incidence of AKP in this meta‐analysis. In the LT subgroup, four studies^4^, ^6^, ^10^, ^11^ reported the incidence of AKP. On meta‐analysis, there was no significant difference in the incidence of AKP between PD and NPD groups (OR 0.51; 95% CI 0.25 to 1.04; *P* = 0.06; *n* = 605; *I* ^2^ = 63%; Fig. 4B). Similar statistical results were obtained with a sensitivity analysis, suggesting the stability of the result of the incidence of AKP in this meta‐analysis.

#### *Visual Analogue Scale for Pain*

In the ST subgroup, two studies^4^, ^13^ reported the outcome of VAS. The results indicated that the VAS was significantly decreased in the PD group (WMD −0.57; 95% CI −1.02 to −0.11; *P* = 0.01; *n* = 310; *I* ^2^ = 0%, Fig. 4C). In the LT subgroup, two studies^4^, ^12^ reported the outcome of VAS. There was no significant difference in the VAS between PD and NPD groups (WMD −0.31; 95% CI −0.82 to 0.19; *P* = 0.22; *n* = 189; *I* ^2^ = 62%; Fig. 4D).

#### *Patellar Score*

In the ST subgroup, two studies^1^, ^4^ reported the outcome of PS. On meta‐analysis, there was no significant difference in PS between PD and NPD groups (WMD 0.12; 95% CI −0.83 to 1.06; *P* = 0.81; *n* = 225; *I* ^2^ = 15%; Fig. 4E). In the LT subgroup, four studies reported the outcome of PS. The results indicated that PD could significantly improve PS during a FU of more than 12 months (WMD 1.01; 95% CI 0.65 to 1.38; *P* < 0.00001; *n* = 399; *I* ^2^ = 31%; Fig. 4F).

### *Secondary Outcome*

The results of the meta‐analysis are presented in Table 3.

#### *Knee Society Score and Knee Society Score – Function Score*

In the ST subgroup, three studies^1^, ^4^, ^14^ reported the KSS outcome. On meta‐analysis, there was no significant difference in the KSS between PD and NPD groups (WMD 0.31; 95% CI −2.82 to 3.45; *P* = 0.84; *n* = 487; *I* ^2^ = 65%). Similar statistical results were obtained with a sensitivity analysis, suggesting the stability of the result of the KSS in this meta‐analysis. Two studies^4^, ^14^ reported the outcome of the Knee Society Score – Function Score (KSSFS). There was no significant difference in the KSSFS between PD and NPD groups (WMD 2.33; 95% CI −0.80 to 5.47; *P* = 0.14; *n* = 387; *I* ^2^ = 0%). In the LT subgroup, four studies^1^, ^10^, ^11^, ^12^ reported the outcome of KSS. The results indicated that PD could significantly improve the outcome of KSS (WMD 1.12; 95% CI 0.10 to 2.14; *P* = 0.03; *n* = 472; *I* ^2^ = 30%). Three studies^10^, ^11^, ^12^ reported the outcome of KSSFS. There was no significant difference in KSSFS between PD and NPD groups (WMD 2.34; 95% CI −0.84 to 5.52; *P* = 0.15; *n* = 372; *I* ^2^ = 60%). Similar statistical results were obtained with a sensitivity analysis, suggesting the stability of the result of KSSFS in this meta‐analysis.

#### *The Western Ontario and McMaster Universities Osteoarthritis Index*

In the ST subgroup, two studies^6^, ^14^ reported the outcome of WOMAC. The results indicated thaf PD could significantly improve the outcome of WOMAC (WMD −4.63; 95% CI −6.49 to −2.77; *P* < 0.00001; *n* = 446; *I* ^2^ = 0%). Two studies^1^, ^14^ reported the outcome of WOMAC – function. There was no significant difference in WOMAC – function between the PD and NPD groups (WMD −2.14; 95% CI −4.59 to 0.31; *P* = 0.09; *n* = 362; *I* ^2^ = 49%). In the LT subgroup, two studies^10^, ^11^ reported the outcome of WOMAC. The results indicated that PD could significantly improve the outcome of WOMAC (WMD −1.41; 95% CI −2.74 to −0.08; *P* = 0.04; *n* = 302; *I* ^2^ = 0%). Two studies^1^, ^10^ reported the outcome of WOMAC – function. There was no significant difference in WOMAC – function between PD and NPD groups (WMD 0.98; 95% CI −2.10 to 4.07; *P* = 0.53; *n* = 302; *I* ^2^ = 39%).

#### *Oxford Knee Score*

In the ST subgroup, two studies^4^, ^13^ reported the outcome of OKS. There was no significant difference in OKS between PD and NPD groups (WMD −0.18; 95% CI −2.92 to 2.57; *P* = 0.90; *n* = 310; *I* ^2^ = 59%).

#### *Knee Range of Motion*

In the ST subgroup, two studies^4^, ^6^ reported the outcome for ROM. The results indicated that the ROM could be significantly improved in the PD group with a FU of less than 12 months (WMD 9.60; 95% CI 0.39 to 18.81; *P* = 0.04; *n* = 309; *I* ^2^ = 96%). In the LT subgroup, three studies^4^, ^11^, ^12^ reported the outcome for ROM. There was no significant difference in the ROM between PD and NPD groups (WMD 2.62; 95% CI −0.52 to 5.75; *P* = 0.10; *n* = 289; *I* ^2^ = 76%).

#### *Complications*

In the ST subgroup, three studies^13^, ^14^, ^15^ reported the outcome of the incidence of complication. There was no significant difference in the incidence of complications between PD and NPD groups (WMD 2.56; 95% CI 0.49 to 13.44; *P* = 0.27; *n* = 425; *I* ^2^ not available). In the LT subgroup, six studies reported the outcome of the incidence of complications. There was no significant difference in the incidence of complications between PD and NPD groups (WMD 0.61; 95% CI 0.30 to 1.24; *P* = 0.18; *n* = 775; *I* ^2^ = 0%).

## Discussion

Anterior knee pain after TKA could be a precursor to low patient satisfaction and bad knee function^16^. Patellar resurfacing is thought to be an effective method to release AKP. However, according to the results of a recent study^17^, the efficacy of patellar resurfacing is imprecise. A study comparing patellar resurfacing and PD showed that there is no significant difference in the incidence of AKP with a 2‐year FU^18^. As patellar resurfacing could result in several complications, such as fracture, loosening, and dislocation^19^, ^20^, many surgeons prefer to not perform patellar resurfacing in TKA. Though not clearly understood, there are many reasons for the AKP, such as infrapatellar fat pad excision, the design and position of the implant, the size and rotation of the trochlear groove, and the surgical technique^21^, ^22^. Theoretically, blocking superficial sensory nerves around the patella could reduce the incidence of AKP and improve the knee function after TKA. Unfortunately, no consensus regarding to the effect of PD in TKA has been achieved. Previous RCT and meta‐analyses have indicated that there is a correlation between the outcomes of PD and FU^5^, ^7^. Thus, to evaluate the outcomes in different periods after the operation, we previously grouped the outcomes according to FU, and data was, respectively, extracted and analyzed for each group to help surgeons decide whether or not to use PD in TKA procedures.

Because of the insufficient data on the depth of PD, subgroup analysis could not be conducted to explore the influence of the depth of PD on AKP and knee function, which is a limitation of this study. We believe that more studies addressing the depth of PD should be conducted.

In this meta‐analysis, we previously grouped the outcomes according to FU and listed VAS, PS, and the incidence of AKP as primary outcomes. Similar to the result of the recent meta‐analysis^7^, PD could reduce the incidence of AKP during FU within 12 months but not during FU after 12 months. In addition, PD could significantly improve PS during FU after 12 months, but not during FU within 12 months. However, the VAS was also improved during FU within 12 months, which is inconsistent with the result of a previous study^7^. This finding might be due to one trial^1^ being excluded because only the VAS for satisfaction but not for pain was presented as a result. To explore the potential sources of heterogeneity, we performed a sensitivity analysis. The results were consistent with previous results, which indicates the stability of our findings.

We also performed a meta‐analysis for KSS, KSSFS, WOMAC, WOMAC – function, OKS, and ROM as secondary outcomes to evaluate the knee function. According to the results, PD could improve WOMAC both within short‐term and long‐term FU. In addition, the ROM of the knee joint was improved during FU within 12 months, and KSS was also improved during FU after 12 months. These results indicated that PD was effective in improving knee function after TKA. This finding was opposite to the result of the meta‐analysis of Xie *et al*.^23^, which might be due to one recent trial being included for more comprehensive analysis and the improved methodology of this study.

Eight trials reported the incidence of complications, but complications associated with TKA, including deep vein thrombosis, persistent ooze, limited ROM needing manipulation, infection, or loosening of implants, were detected in just three trials. Theoretically, PD might interrupt the blood supply to the patella and induce patellar osteonecrosis^24^, ^25^. However, no patellar osteonecrosis was detected in all included trials. The meta‐analysis result showed that PD did not increase the incidence of complications, which indicated that PD was a safe technique in TKA.

This meta‐analysis has several strengths. First, a comprehensive database was researched and nine RCT were included. Involving 1065 knees, this study, to our knowledge, has a larger sample size than previous meta‐analyses, which may lead to more veritable conclusions. In addition, more indicators were used to assess AKP and knee function than in previous meta‐analyses. Finally, to explore the potential sources of heterogeneity, a sensitivity analysis was performed, which suggested the stability of the conclusions in this meta‐analysis.

In conclusion, current evidence suggests that PD could significantly reduce the VAS and the incidence of AKP in FU within 12 months and improve the short‐term and long‐term knee function. Compared with NPD, PD is a safe and recommendable technique that could be routinely performed in TKA.
